# Entry of Panton–Valentine leukocidin-positive methicillin-resistant *Staphylococcus aureus* into the hospital: prevalence and population structure in Heidelberg, Germany 2015–2018

**DOI:** 10.1038/s41598-020-70112-z

**Published:** 2020-08-06

**Authors:** Sabrina Klein, Julius Hannesen, Philipp Zanger, Klaus Heeg, Sébastien Boutin, Dennis Nurjadi

**Affiliations:** 1 Medical Microbiology and Hygiene, Department of Infectious Diseases, Heidelberg University Hospital, Im Neuenheimer Feld 324, 69120 Heidelberg, Germany; 2 Heidelberg Institute of Global Health, Heidelberg University Hospital, Heidelberg, Germany

**Keywords:** Clinical microbiology, Infectious-disease epidemiology

## Abstract

*Staphylococcus aureus* is one of the major pathogens causing community—and healthcare-acquired infections. The presence of the virulence factor Panton–Valentine leukocidin (PVL) is associated with recurrent infection and clinical severity and generally regarded as a feature of community associated-methicillin-resistant *Staphylococcus aureus* (MRSA). To date, the focus of PVL-positive MRSA in hospitalized patients has been on outbreaks. We aimed to investigate whether PVL-positive MRSA has penetrated the community-hospital barrier by determining the prevalence of PVL in MRSA of hospitalized patients. MRSA strains isolated from patients hospitalized > 48 h in Heidelberg University Hospital between 2015 and 2018 Isolates were analysed for the presence of PVL and subjected to *spa*-typing. PVL-positive MRSA were then characterized by whole genome sequencing. We analysed 740 MRSA isolates in the study period and identified 6.2% (n = 46) PVL-positivity. 32.6% of PVL-positive MRSA met the criteria for nosocomial acquisition. The most frequent clones among the PVL-positive strains were ST80-t044 (21.7%, n = 10/46) and ST8-t008 (19.5%, n = 9/46). WGS identified three possible transmission clusters involving seven patients. In conclusion, we found successful epidemic PVL-positive MRSA clones entering the hospital and causing nosocomial infections. Preventive measures and constant surveillance should be maintained to prevent transmissions and clonal outbreaks.

## Introduction

*Staphylococcus aureus* is one of the major causes of community and hospital acquired infections. Methicillin-resistance is an ongoing problem locally and globally. Although the overall prevalence of methicillin-resistant *S. aureus* (MRSA) in Germany is declining^1^^,^ MRSA strains harboring the pathogenic marker Panton–Valentine leukocidin (PVL) are isolated more frequently^2^. The presence of PVL is relevant, as it is associated with a more severe clinical presentation, often with recurrences, deep and multiple lesions and frequent transmission to contact persons, irrespective of methicillin resistance^3–6^. In addition, PVL-positive *S. aureus* often displays multiple resistances to commonly used antibiotics to treat *S. aureus* infections^3,7^.

PVL has been linked to community-associated (CA-) MRSA^6^. Although the overall prevalence of PVL in Germany is considered low according to data acquired by Schaumburg et al.^8^, a study by Jappe et al.^9^ revealed a prevalence of 22% for PVL positivity in MRSA from a dermatological outpatient clinic from 2003 to 2005. Moreover, our recent study suggests an alarmingly high prevalence of PVL positivity (40%) in community onset (CO)-MRSA causing skin and soft-tissue infections (SSTI) in South West Germany between 2012 and 2016^2^. Nevertheless, systematic data on the prevalence and molecular characteristics of PVL-bearing MRSA in hospitalized patients in Germany is scarce. Surveillance data and other published report suggest that a high percentage of imported epidemic MRSA clones are circulating and causing community acquired infections^2–4^. More importantly, patients with CO-MRSA SSTI often seek medical care in out-patient units and emergency units of tertiary hospitals^10,11^, providing a port of entry for these MRSA clones to the hospital setting.

In this study, we analyzed and characterized MRSA isolates from hospitalized patients with a special focus on PVL-positive MRSA by WGS retrospectively to understand the population structure and dynamics of PVL positive MRSA entering and circulating in our hospital. Clonal shift of MRSA population in the healthcare setting should be monitored closely to prevent clonal spread of highly virulent and epidemic MRSA clones.

## Results

Between 2015 and 2018 we detected and isolated 756 non-duplicate MRSA from overall 756 individual patients admitted to Heidelberg University hospital with 188, 158, 210 and 184 isolates in the respective years (Table 1). 58.1% (n = 439/756) patients were male. The mean age was 52 years with a range from 3 days to 97 years.

**Table 1:** Prevalence of PVL in MRSA isolated from hospitalized patient, Germany 2015–2018.

Year	Total MRSA	PVL positive		PVL negative	
	n	n	%	n	%
2015	188	5	2.7	183	97.3
2016	158	17	10.8	141	89.2
2017	210	13	6.2	197	93.8
2018	184	11	6.0	173	94.0
Total	740	46	6.2	694	93.8

### Molecular characteristics of MRSA from hospitalized patients

Of 756 MRSA, 22 isolates were not recoverable for further molecular characterization by *spa*-typing and WGS. The most common *spa* types (> 20 isolates) found in our MRSA collection were t003 (31.6%; n = 232/734), t002 (6.0%; n = 44/734), t127 (5.6%; n = 41/734), t008 (4.9%; n = 36/734) and t063 (2.7%; n = 20/734) (for all *spa*-types, see supplemental Table). The majority of characterized MRSA harboured SCC*mec* types II and IV (n = 326 and n = 301; 43.1% and 39.9% respectively). All MRSA isolates harboured *mecA*.

For 740 of 756 MRSA, data regarding the presence of PVL was available. Of 740 isolates, 46 (6.2%) were PVL-positive with 2.7% (n = 5/188), 10.8% (n = 17/158), 6.2% (n = 13/210) and 6.0% (n = 11/184) in the respective years (Table 1).

### Clinical and molecular characteristics of PVL-positive MRSA

**Figure 1:**
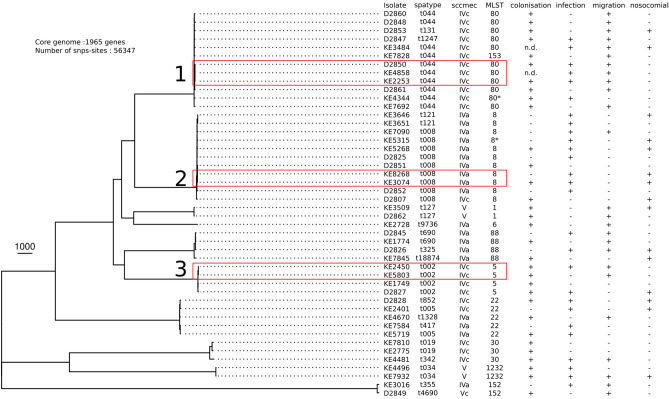
Phylogenetic tree of PVL-positive MRSA. Strains with a SNP distance lower that 10 SNPs are highlighted by a red rectangle. These were potential in-hospital transmission cases between patients (named cluster 1, 2 and 3).

We identified 46 (6.2%) patients with PVL-positive MRSA in the study period (Table 1). Most patients were males (58.7%, n = 27/46) and mean age was 36 years with a range of 1 month to 96 years. Patients with PVL-positive MRSA were significantly younger than those with PVL-negative MRSA (mean age 36 years and 53 years, respectively, *p* < 0.0001). The proportion of males did not differ significantly between the two groups (*p* = 0.93). 58.7% (n = 27/46) of the patients had an infection with PVL-positive MRSA, of which 69.6% (n = 32/46) were simultaneously colonized while 26% (n = 12/46) were free of nasal and rectal PVL-positive MRSA on screening swabs. Two patients with PVL-positive MRSA infection were not screened for rectal or nasal MRSA colonization. 54.3% (n = 25/46) of patients had a documented history of migration. 15 patients (32.6%) met the criteria for nosocomial MRSA acquisition of being detected > 48 h after hospital admission (Fig. 1). Thus, the majority of patients (67.4%, n = 31/46) was already infected or colonized with PVL-positive MRSA on admission to hospital. Skin and soft tissue infections were the most common clinical presentation (68.8% of cases, multiple clinical presentations possible). Two cases presented with blood stream infections and two with bone and joint infections (Table 2).

**Table 2:** Site of infection in patients with PVL-positive MRSA

Site of infection	n	%
Skin and soft tissue	22	68.8
Respiratory tract	3	9.4
Blood stream	2	6.3
Bone and joint	2	6.3
Other	3	9.4

Multiple sites of infection possible.

Among the PVL-positive MRSA, we identified 10 clonal groups according to MLST. These were ST1, ST5, ST6, ST8, ST22, ST30, ST80, ST88, ST152 and ST1232 (Fig. 1), each representing a genetic cluster. The *spa*-types identified were for ST1 t127, for ST5 t002, for ST6 t9736, for ST8 t008 and t121. With ST22, *spa*-type t852, t005, t1328 and t417 were present. For ST30, t019 and t342 and for ST80 t044, t131 and t1247 were identified. *Spa*-type t690, t325 and t18874 of ST88 were detected and with ST152, t355 and t4690 were identified. With ST1232, *spa*-type t034 was detected. The most prevalent spa types were t044 (n = 10/46; 21.7%) and t008 (n = 9/46; 19.5%). The PVL-positive isolates were of the SCC*mec* type IV and V (Fig. 1). The most common *spa* type causing infections was t008 (n = 7/27; 25.9%), followed by t044 (n = 5/27; 18.5%).

Among the hospital onset PVL-positive MRSA (n = 15/46), ST8 (t008 and t121) MRSA was the most common, accounting for 40% (n = 6/15). The majority of these isolates (n = 5/6, 83%) belonged to the USA300 clonal group (ST8-t008-IVa/c). Other common PVL-positive MRSA clones were ST80-t044, ST88 and ST22 with two isolates each, and ST1-t127, ST5-t002 and ST1232-t034 with one isolates each (Fig. 1).

### Phenotypical antimicrobial resistance and detected resistance genes in PVL-positive MRSA

**Figure 2:**
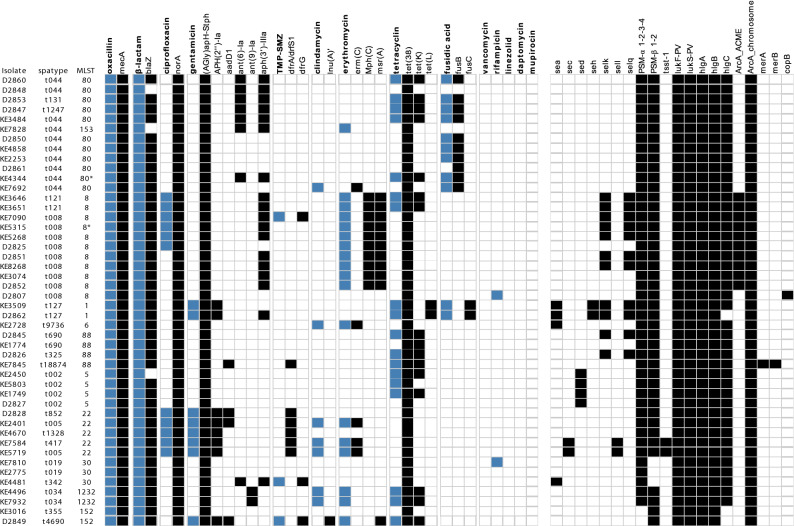
Phenotypic and genotypic antimicrobial resistance of PVL-positive MRSA. Phenotypic resistance is represented by a blue square while genotypic resistance is represented by a black square. The right panel represents the virulence genes and ACME/COMER cassettes found in the strains. Strains are ordered based on the phylogenetic tree from Fig. 1.

Of the PVL-positive MRSA, 23.9% (n = 11/46; 3 missing data) were resistant to ciprofloxacin, belonging to ST22 (5/11) and ST8 (6/11) (Fig. 2). 17.4% (n = 8/46) were resistant to gentamicin, had all the *APH*(2″)-*Ia* gene, and belonged either to ST22, ST1 or ST152. Co-trimoxazole (TMP-SMZ) resistance was not common: only 6.5% (n = 3/46) of PVL-positive MRSA were resistant, all of which carried *dfrG*. Phenotypical resistance to clindamycin in 15.2% (n = 7/46) did not correlate to the presence of *Inu(A)′*. 39.1% (n = 18/46) were resistant to erythromycin. *Erm(C)* was present in 5 strains, of which 4 were phenotypically resistant. 10 strains carried *Mph(C)* and *msr(A),* were all phenotypically resistant and belonged to the ST8 (8 *spa*-type t008 and 2 *spa*-type t121). Only one strain with resistance to erythromycin harboured the *msr(A)* gene alone (ST152). Resistance to tetracycline was present in 39.1% (n = 18/46), 15 of these carried the *tet(K)* and two the *tet(L)* gene. Fusidic acid resistance was found in 23.9% (n = 11/46) of isolates, 9 of which carried the *fusB* gene; all of them belonging to the ST80. Two (4.3%) carried the *fusC* gene and belonged to ST1, *spa*-type t127. Two PVL-positive MRSA (ST8-t008 and ST30-t019) were resistant to rifampicin and carried functionally related mutations in the *rpoB* gene. Neither phenotypical resistance towards vancomycin, linezolid, daptomycin or mupirocin, nor any corresponding resistance gene was detected. For all detected resistance genes, see Fig. 2.

### Virulence genes present in PVL-positive MRSA

All Isolates harboured genes encoding phenol soluble modulins, 95% (n = 44/46) of them for PSM-α 1–4 and PSM-ß 1–2. Two isolates of ST152 carried only genes for PSM-ß 1–2 and three strains of ST30 only for PSM-α 1–4 (Fig. 2). Likewise, *hlgA* and *hlgB* were present in all strains, and *hlgC* was not detected in only three strains, both of ST152 and one of ST1 (t127). 10 of the isolates belonging to ST8 (SCC*mec* IVa) harboured the arginine catabolic mobile element (ACME), a hallmark of ST8-USA300 MRSA. The other, ACME-negative, ST8 t008 (SCC*mec* IVc) strain displayed the *copB* gene as part of the copper and mercury resistance mobile element (COMER), described as a key feature of the Latin American Variant (USA300-LV). In 7 of the ST8 (t008 and t121) *selK* and *selq* was detected. Two strains of ST22 were positive for *tsst-1*, one with *spa*-type t005 and the other with t417. For all genes encoding virulence factors, see Fig. 2.

### Possible transmission events

SNP analysis identified three possible transmission clusters involving 7 patients (Fig. 1, red rectangles). These included three patients with ST80 (t044) in cluster 1, two patients with ST8 (t008) in cluster 2 and another two patients with ST5 (t002) in cluster 3. All three patients with t044 had overlapping hospitals stays on the same wards. The two patients with t008 and both patients with t002 had no temporal or spatial (same ward) overlap during their hospital stay.

## Discussion

PVL is regarded as one of the main features of CA-MRSA and a clinically relevant virulence marker for *S. aureus*^3^. In recent years, cases of nosocomial PVL-positive MRSA transmissions and outbreaks in Europe have been accumulating^5,12,13^ thus suggesting that the barrier between community- and hospital-associated MRSA has already been penetrated. Although PVL is associated with the severity of infection^3^^,^ the characterization of MRSA isolates in hospitalized patients in Germany in terms of PVL is not consequently pursued. To our knowledge, this is the first systematic analysis on the prevalence and molecular structure of PVL-positive MRSA in hospitalized patients in Germany.

We found that the clonal distribution as determined by MLST and *spa*-typing was overall concordant with existing knowledge about the molecular epidemiology of MRSA in Germany: 30.5% of isolates were *spa*-type t003, consistent with the predominant ST5 “Rhine-Hesse epidemic clone”^14^ and 5.8% t002^8^. Likewise ST8 t008 MRSA (4.6%,n = 35/756) is considered one of the most common MRSA clones circulating in Germany^8^. Unexpectedly, our sample contained 2.6% (n = 20/756) of t063 MRSA which has been rarely described in Germany, yet^8^. This was explained by a putative transmission cluster of PVL-negative MRSA in our hospital.

Altogether, we detected 6.2% PVL positivity in MRSA. This was considerably higher that the findings of another study on MRSA prevalence in hospitalized and non-hospitalized patients in Germany 2010–2011, which reported 2.7% being PVL-positive^8^. A possible explanation for this discrepancy could the premise of the analysis. The authors did not differentiate between in-patients and out-patients for their analysis, it is therefore difficult to directly compare the reported rates of PVL-positive MRSA between the two studies. Similar to our findings, a Swiss study reported 7.7% PVL-positive MRSA in a mixed population of hospitalized and non-hospitalized patients^15^. Similarly, a European study conducted among patients presenting with skin and soft tissue infections in the emergency room reported 8% PVL-positive MRSA for the German study centre^10^. Compared to the 40% prevalence of PVL-positive MRSA in patients presenting with SSTI in outpatient departments of our hospital^2^, these estimates and our findings support, that only a fraction of these patients actually enter the hospital. However, we also found PVL-positive MRSA in blood cultures and tissue samples from bone and joint infections, providing evidence for invasive infections in this population besides the high percentage of skin and soft tissue infections.

Over two thirds (67.4%) of patients with PVL-positive MRSA in our study were already colonized or infected on hospital admission. This was somewhat expected, since the presence of PVL has been linked to community-associated clones^2,16^. In line with our previous findings on the genetic background of MRSA clones in community onset SSTI, the majority of characterized isolates in this study belonged to ST8-t008 and ST80-t044^2^. ST80-t044 MRSA clonal lineage has been described as a frequent CA-MRSA clone circulating in Europe and the Middle East^17,18^. Another dominant clone was the ST8-t008 MRSA belonging to the epidemic USA300 clade (both North-American and Latin American variant). Although the prevalence of this MRSA clone in Europe is generally considered to be low^8,10^, we found a remarkably high proportion of USA300 MRSA clones circulating in the community^2^ and USA300 is indeed the most commonly imported MRSA into the European continent through intercontinental travel^19^. Moreover, USA300 MRSA clones can be classified as epidemic, as demonstrated by their expansive properties and dominance on the American continents^20^ and further highlighted by accumulating reports on nosocomial transmission events and outbreaks in Europe^5,12,13^.

Another interesting clone is the ST1-t127 MRSA, which is regarded as livestock-associated MRSA (LA-MRSA), made up 5.4% of our study isolates. This particular clone has been linked with bovine mastitis and colonization of farm animals. Although several reports of human infections with ST1-t127 MRSA have been published, this occurrence is still considered rare^21,22^. Moreover, some of the LA-MRSA clones harbor the virulence factor PVL. Although reports on the presence of PVL genes in ST1-t127 have been published, the isolates in those reports harbour the human-adapted SCCmec IV^22^. In contrast our PVL-positive ST1-t127 MRSA were of the SCCmec V, consistent with the porcine ST1-t127 MRSA^22^. The acquisition of PVL was not only restricted to the ST1 clones but several LA-MRSA isolates with ST1232 (t034), a single locus variant of the ST398 LA-MRSA, suggesting human adaptation of LA-MRSA clones^22^. Taken together, these finding demonstrates the relevance of the “One Health” concept for the surveillance of multi-drug resistance bacteria.

Of the three previously undetected potential transmission clusters identified by SNP analysis, one cluster most likely represents nosocomial transmission events, since these three patients had overlapping hospital stays on the same ward (t044, cluster 1, Fig. 1). The two patients with t008 MRSA (cluster 2, Fig. 1) had no temporal or spatial overlap. Comparative analysis of virulence genes revealed slight differences in genes present/absent (Fig. 2), suggesting non-identical isolates despite close clonal relationship. Therefore, patient-to-patient transmission was unlikely. In the third potential transmission cluster (t002, cluster 3, Fig. 1), both patients did not have apparent epidemiological overlap during their hospital stays. However, since both patients were housed in the same residential facility for refugees, a transmission event outside of the hospital setting is a plausible alternative explanation. Our findings underline the importance of reliable epidemiological data for investigations of transmissions and outbreaks of multi-drug resistant bacteria.

Our study has strengths and limitations. It was a single-centre study and may therefore only reflect the local epidemiological situation in a part of Germany. However, this systematic analysis provides evidence on the occurrence of PVL-positive MRSA over a 4-year period in a tertiary care hospital. Molecular typing data have indicated that epidemic PVL-positive MRSA clones have entered the hospital and may cause nosocomial infections and outbreaks. Thus, implementation of pre-emptive measures, such as systematic surveillance of MRSA population structures in hospitalized and non-hospitalized patients is needed, with special emphasis on PVL-positive clones.

## Methods

### Study population

We analysed and characterized MRSA isolates from hospitalized patients in our tertiary care hospital over a 4-year period between 2015 and 2018 retrospectively. The study was performed at Heidelberg University Hospital, located in the South-West of Germany. Clinical and microbiological data were acquired though the routine microbiological diagnostic, surveillance of multidrug resistant bacteria and patient care. Only hospitalized patients (> 48 h) were included in this study.

### Microbiological methods

MRSA were cultured and detected from routine screening samples and clinical samples from hospitalized patients seeking medical care, as described previously^2^. Species identification was performed with the MALDI-TOF (Bruker GmbH, Germany), antibiotic susceptibility testing using the VITEK2 (Biomérieux GmbH, Germany) and interpreted according to current EUCAST clinical breakpoints. MRSA was confirmed by the presence of *nuc* and *mecA* as described earlier^2^. Isolates were cryopreserved for surveillance purposes.

### Spa typing, SCCmec typing and detection of PVL

PVL detection was performed by PCR, as previously described^3^. *Spa* typing was performed by Sanger sequencing as published elsewhere^23^. SCC*mec* typing for the cassette types I to V were determined by multiplex PCR as described earlier^24^. All PVL positive strains were subject to WGS for a more detailed analysis.

### WGS

Genomic DNA was extracted from overnight bacterial culture using the DNeasy Blood and Tissue Minikit (Qiagen GmbH, Germany) according to manufacturer’s protocol with the addition of a prior lysis step with lysostaphin (Genaxxon GmbH, Germany). Standard genomic library was prepared from the DNA and sequenced with the Illumina MiSeq platform (2 × 250 bp paired end). Quality control and assembly was performed as described previously^25^.

Briefly, raw sequences were trimmed for quality using Sickle 1.33 (parameters, q > 30; 1 > 45)^6^. The cleaned sequences were then assembled using SPAdes 3.13.0^7^. Contigs obtained from the assembly were curated for length (> 500 bp) and coverage (> 10 ×) to ensure no errors and contamination in the draft genome. Annotation was performed using Prokka 1.14.1 (based on Genetic Code Table 11)^8^ and the NCBI Prokaryotic Genome Annotation Pipeline. Resistance and virulence genes were found using Abricate 0.8.13 (https://github.com/tseemann/abricate with respectively the antibiotic database from ResFinder 3.0; ARG-ANNOT; CARD; NCBI-BARRGD and the virulence database VFDB. MLST was perform using the MLST 2.0 pipeline from the centre for Genomic Epidemiology^26^. Sequences will be made publically available upon publication of the manuscript.

### Cluster analysis by SNP and allelic difference

Genomes were compared by calculating the core genomes with Roary and only genes present in all isolates were considered (1965 genes, 74.7 ± 1.7% of the genomes). Phylogenetic distance was calculated with Gubbins 2.3.4 to take in account recombination events and not over-estimate the SNP polymorphism^27^. Clonal groups were defined as genomes distant from less than 10 SNPs.

### Data and statistical analysis

Patient data was extracted from the medical record. A history of migration was defined as a patient with home address outside of Germany or living in a community facility for refugees. Descriptive statistics was analyzed using the STATA 13 software (StataCorp, USA). Two-sample *t* test or chi square test was used calculating the *p* value for age and sex, *p* < 0.05 was considered statistically significant.

### Ethical considerations

Ongoing molecular characterization of MRSA isolates is performed as part of obligatory surveillance and infection control measures mandated by the German Infection Protection Act. All methods were carried out in accordance with relevant guidelines and regulations. The Ethical review board of the University of Heidelberg approved the study protocol (S-474/2018) and waived informed consent.

## Supplementary information

Supplementary Information.

## Data Availability

Sequences were uploaded to https://www.ncbi.nlm.nih.gov/biosample with the BioProject Number PRJNA637212. Accession numbers are listed in Supplementary Table 3.
