# Supplementary material for: Entry of Panton–Valentine leukocidin-positive methicillin-resistant Staphylococcus aureus into the hospital: prevalence and population structure in Heidelberg, Germany 2015–2018
Source: Sci Rep. 2020 Aug 6;10:13243. doi: 10.1038/s41598-020-70112-z (PMC7413528; doi:10.1038/s41598-020-70112-z)
Supplement: Supplementary file 1 — Supplementary Information. [file 41598_2020_70112_MOESM1_ESM.docx]

**Supplementary materials**

**Table 1 *Spa*- types of all MRSA strains in hospitalized patients, 2015-2018.**

| ***spa*-type** | **n** |
| --- | --- |
| t003 | 232 |
| t002 | 44 |
| t127 | 41 |
| t008 | 36 |
| t063 | 20 |
| t223 | 18 |
| t032 | 16 |
| t608 | 16 |
| t044 | 13 |
| t045 | 13 |
| t011 | 11 |
| t16990 | 10 |
| t267 | 9 |
| t005 | 8 |
| t304 | 8 |
| t034 | 7 |
| t264 | 7 |
| t021 | 6 |
| t1107 | 5 |
| t1227 | 5 |
| t728 | 5 |
| t088 | 4 |
| t439 | 4 |
| t504 | 4 |
| t010 | 3 |
| t014 | 3 |
| t037 | 3 |
| t091 | 3 |
| t16333 | 3 |
| t1947 | 3 |
| t437 | 3 |
| t688 | 3 |
| t843 | 3 |
| t015 | 2 |
| t019 | 2 |
| t030 | 2 |
| t105 | 2 |
| t11791 | 2 |
| t121 | 2 |
| ***spa*-type** | **n** |
| t13454 | 2 |
| t15422 | 2 |
| t16128 | 2 |
| t1779 | 2 |
| t1814 | 2 |
| t242 | 2 |
| t321 | 2 |
| t3212 | 2 |
| t325 | 2 |
| t362 | 2 |
| t386 | 2 |
| t390 | 2 |
| t463 | 2 |
| t535 | 2 |
| t685 | 2 |
| t690 | 2 |
| t7784 | 2 |
| t790 | 2 |
| t9736 | 2 |
| t020 | 1 |
| t024 | 1 |
| t035 | 1 |
| t040 | 1 |
| t050 | 1 |
| t062 | 1 |
| t067 | 1 |
| t073 | 1 |
| t084 | 1 |
| t1048 | 1 |
| t1083 | 1 |
| t10963 | 1 |
| t11475 | 1 |
| t1198 | 1 |
| t1247 | 1 |
| t131 | 1 |
| t1328 | 1 |
| t13308 | 1 |
| t137 | 1 |
| t13748 | 1 |
| ***spa*-type** | **n** |
| t1442 | 1 |
| t1451 | 1 |
| t1476 | 1 |
| t14825 | 1 |
| t15088 | 1 |
| t151 | 1 |
| t15173 | 1 |
| t15604 | 1 |
| t15605 | 1 |
| t15636 | 1 |
| t15686 | 1 |
| t1623 | 1 |
| t16328 | 1 |
| t16330 | 1 |
| t16331 | 1 |
| t16651 | 1 |
| t174 | 1 |
| t1767 | 1 |
| t1771 | 1 |
| t17983 | 1 |
| t17984 | 1 |
| t18096 | 1 |
| t18097 | 1 |
| t1842 | 1 |
| t186 | 1 |
| t18874 | 1 |
| t190 | 1 |
| t1910 | 1 |
| t214 | 1 |
| t216 | 1 |
| t2183 | 1 |
| t2319 | 1 |
| t2365 | 1 |
| t2518 | 1 |
| t2571 | 1 |
| t2576 | 1 |
| t2849 | 1 |
| t309 | 1 |
| t3092 | 1 |
| t311 | 1 |
| t3195 | 1 |
| t3209 | 1 |
| t3219 | 1 |
| t3297 | 1 |
| t342 | 1 |
| t3507 | 1 |
| ***spa*-type** | **n** |
| t3515 | 1 |
| t355 | 1 |
| t359 | 1 |
| t3841 | 1 |
| t4000 | 1 |
| t417 | 1 |
| t4298 | 1 |
| t4324 | 1 |
| t4450 | 1 |
| t449 | 1 |
| t451 | 1 |
| t456 | 1 |
| t4690 | 1 |
| t4712 | 1 |
| t481 | 1 |
| t5076 | 1 |
| t5121 | 1 |
| t515 | 1 |
| t5168 | 1 |
| t5379 | 1 |
| t541 | 1 |
| t547 | 1 |
| t548 | 1 |
| t5507 | 1 |
| t564 | 1 |
| t6076 | 1 |
| t620 | 1 |
| t626 | 1 |
| t627 | 1 |
| t6449 | 1 |
| t692 | 1 |
| t7383 | 1 |
| t748 | 1 |
| t7752 | 1 |
| t786 | 1 |
| t788 | 1 |
| t852 | 1 |
| t887 | 1 |
| t9205 | 1 |
| t9606 | 1 |
| t982 | 1 |
| t991 | 1 |
| other | 4 |
| n.t. | 3 |
| non recoverable | 22 |
|  |  |

Supplementary table 1: *Spa-* types of all MRSA isolates from hospitalized patients in Heidelberg University Hospital 2015-2018. 22 strains were not recoverable from cryostock. Abbr.: n.t.: non-typable.

**Table 2 Assembly description of all MRSA strains in hospitalized patients, 2015-2018.**

| **Isolate** | **coverage** | **# contigs** | **Largest contig (bp)** | **Total length (bp)** | **GC content (%)** | **N50** | **N75** | **L50** | **L75** |
| --- | --- | --- | --- | --- | --- | --- | --- | --- | --- |
| D2807 | 34 | 18 | 911927 | 2866043 | 32,66 | 610571 | 399214 | 2 | 4 |
| D2825 | 31 | 26 | 881111 | 2858640 | 32,63 | 345572 | 207197 | 3 | 5 |
| D2826 | 40 | 16 | 718683 | 2843634 | 32,67 | 346180 | 245644 | 3 | 5 |
| D2827 | 36 | 16 | 775239 | 2785973 | 32,7 | 717311 | 242057 | 2 | 5 |
| D2828 | 41 | 44 | 239137 | 2764065 | 32,74 | 150780 | 92651 | 8 | 14 |
| D2845 | 38 | 21 | 718806 | 2833126 | 32,68 | 346391 | 170514 | 3 | 5 |
| D2847 | 35 | 23 | 737240 | 2807064 | 32,71 | 334344 | 143584 | 3 | 7 |
| D2848 | 36 | 16 | 894717 | 2776647 | 32,73 | 322145 | 296425 | 3 | 5 |
| D2849 | 32 | 34 | 790903 | 2805501 | 32,62 | 326249 | 100070 | 3 | 7 |
| D2850 | 41 | 16 | 881403 | 2765897 | 32,73 | 394831 | 208385 | 3 | 5 |
| D2851 | 114 | 21 | 881738 | 2879561 | 32,62 | 378663 | 180701 | 3 | 5 |
| D2852 | 39 | 22 | 749036 | 2906192 | 32,66 | 472946 | 129260 | 3 | 5 |
| D2853 | 114 | 17 | 1005110 | 2806740 | 32,72 | 430661 | 381829 | 2 | 4 |
| D2860 | 31 | 21 | 630002 | 2794101 | 32,73 | 319850 | 207054 | 4 | 6 |
| D2861 | 128 | 13 | 880692 | 2766985 | 32,73 | 662372 | 229968 | 2 | 4 |
| D2862 | 117 | 36 | 331284 | 2859318 | 32,67 | 202000 | 99655 | 6 | 11 |
| KE1749 | 44 | 22 | 930712 | 2792410 | 32,71 | 638843 | 136658 | 2 | 5 |
| KE1774 | 36 | 20 | 974421 | 2829219 | 32,69 | 718817 | 530618 | 2 | 3 |
| KE2253 | 67 | 14 | 881403 | 2767083 | 32.73 | 394831 | 208385 | 3 | 5 |
| KE2401 | 40 | 61 | 244276 | 2806286 | 32,75 | 101411 | 54740 | 9 | 19 |
| KE2450 | 47 | 19 | 775253 | 2789318 | 32,69 | 715496 | 199238 | 2 | 5 |
| KE2728 | 48 | 17 | 872866 | 2832386 | 32,74 | 832283 | 545350 | 2 | 3 |
| KE2775 | 37 | 29 | 741831 | 2781042 | 32,71 | 249623 | 124822 | 3 | 8 |
| KE3016 | 35 | 48 | 618547 | 2820328 | 32,66 | 250619 | 83916 | 4 | 9 |
| KE3074 | 36 | 27 | 867781 | 2861890 | 32,63 | 345191 | 145653 | 3 | 6 |
| KE3484 | 38 | 21 | 879832 | 2830880 | 32,75 | 381606 | 208385 | 3 | 5 |
| KE3509 | 36 | 27 | 406553 | 2814539 | 32,64 | 271076 | 118504 | 5 | 9 |
| KE3516 | 39 | 19 | 868263 | 2834754 | 32,66 | 374168 | 283444 | 3 | 5 |
| KE3646 | 29 | 29 | 938354 | 2920304 | 32,65 | 345572 | 159044 | 3 | 6 |
| KE3651 | 36 | 22 | 1284231 | 2919493 | 32,64 | 429984 | 173227 | 2 | 4 |
| KE4344 | 38 | 23 | 836975 | 2794252 | 32,73 | 377442 | 276218 | 3 | 5 |
| KE4481 | 53 | 48 | 174096 | 2847525 | 32,76 | 109597 | 66809 | 11 | 19 |
| KE4496 | 41 | 26 | 340663 | 2813921 | 32,8 | 204059 | 170282 | 6 | 9 |
| KE4670 | 39 | 40 | 402372 | 2756141 | 32,73 | 156529 | 93081 | 6 | 12 |
| KE4858 | 94 | 18 | 711622 | 2781276 | 32.72 | 394831 | 151866 | 3 | 6 |
| KE5268 | 24 | 25 | 895306 | 2875931 | 32,62 | 310176 | 173225 | 3 | 6 |
| KE5315 | 37 | 21 | 1240997 | 2834019 | 32,62 | 406142 | 226479 | 2 | 4 |
| KE5719 | 23 | 51 | 187447 | 2789755 | 32,73 | 124128 | 89833 | 9 | 16 |
| KE5803 | 42 | 17 | 775240 | 2746662 | 32,68 | 715507 | 199248 | 2 | 5 |
| **Isolate** | **coverage** | **# contigs** | **Largest contig (bp)** | **Total length (bp)** | **GC content (%)** | **N50** | **N75** | **L50** | **L75** |
| KE7090 | 45 | 22 | 881520 | 2924128 | 32,65 | 382892 | 250551 | 3 | 5 |
| KE7584 | 40 | 43 | 188114 | 2818099 | 32,72 | 121404 | 89833 | 10 | 17 |
| KE7692 | 41 | 16 | 1005454 | 2770772 | 32,71 | 612811 | 170502 | 2 | 4 |
| KE7810 | 31 | 27 | 641807 | 2775699 | 32,71 | 391221 | 109698 | 3 | 7 |
| KE7828 | 37 | 17 | 837388 | 2782634 | 32,74 | 320201 | 151865 | 3 | 6 |
| KE7845 | 90 | 32 | 625830 | 2865733 | 32,71 | 284466 | 158125 | 4 | 7 |
| KE7932 | 35 | 22 | 723846 | 2776431 | 32,78 | 410573 | 169268 | 3 | 6 |
| KE8268 | 60 | 24 | 881820 | 2876266 | 32.62 | 365195 | 159078 | 3 | 6 |

**Table 3 BioSample Accession numbers of all sequenced isolates**

| **Accession** | **Sample Name** |
| --- | --- |
| SAMN15098634 | D2848 |
| SAMN15098635 | D2860 |
| SAMN15098636 | D2853 |
| SAMN15098637 | D2847 |
| SAMN15098638 | KE3484 |
| SAMN15098639 | KE7828 |
| SAMN15098640 | KE4858 |
| SAMN15098641 | KE2253 |
| SAMN15098642 | D2850 |
| SAMN15098643 | D2861 |
| SAMN15098644 | KE4344 |
| SAMN15098645 | KE7692 |
| SAMN15098646 | KE3646 |
| SAMN15098647 | KE3651 |
| SAMN15098648 | KE7090 |
| SAMN15098649 | KE5268 |
| SAMN15098650 | KE5315 |
| SAMN15098651 | KE3074 |
| SAMN15098652 | D2851 |
| SAMN15098653 | KE8268 |
| SAMN15098654 | D2825 |
| SAMN15098655 | D2852 |
| SAMN15098656 | D2807 |
| SAMN15098657 | D2862 |
| SAMN15098658 | KE3509 |
| SAMN15098659 | KE2728 |
| SAMN15098660 | KE1774 |
| SAMN15098661 | D2845 |
| SAMN15098662 | D2826 |
| SAMN15098663 | KE7845 |
| SAMN15098664 | D2827 |
| SAMN15098665 | KE1749 |
| SAMN15098666 | KE5803 |
| SAMN15098667 | KE2450 |
| SAMN15098668 | KE2401 |
| SAMN15098669 | D2828 |
| SAMN15098670 | KE4670 |
| SAMN15098671 | KE5719 |
| SAMN15098672 | KE7584 |
| SAMN15098673 | KE7810 |
| SAMN15098674 | KE2775 |
| SAMN15098675 | KE4481 |
| SAMN15098676 | KE7932 |
| SAMN15098677 | KE4496 |
| SAMN15098678 | D2849 |
| SAMN15098679 | KE3016 |
|  |  |
